# Role of Long Noncoding RNAs in Parkinson's Disease: Putative Biomarkers and Therapeutic Targets

**DOI:** 10.1155/2020/5374307

**Published:** 2020-06-12

**Authors:** Qiankun Lv, Ziyu Wang, Zhen Zhong, Wei Huang

**Affiliations:** Department of Neurology, The Second Affiliated Hospital of Nanchang University, Nanchang, China

## Abstract

Parkinson's disease (PD) is a neurodegenerative disease characterized by bradykinesia, rigidity, and tremor. Age is the main risk factor. Long noncoding RNAs (lncRNAs) are novel RNA molecules of more than 200 nucleotides in length. They may be involved in the regulation of many pathological processes of PD. PD has a variety of pathophysiological mechanisms, including alpha-synuclein aggregate, mitochondrial dysfunction, oxidative stress, calcium homeostasis, axonal transport, and neuroinflammation. Among these, the impacts of lncRNAs on the pathogenesis and progression of PD need to be highlighted. lncRNAs may serve as putative biomarkers and therapeutic targets for the early diagnosis of PD. This study aimed to investigate the role of lncRNAs in various pathological processes of PD and the specific lncRNAs that might be used as putative diagnostic biomarkers and therapeutic targets of PD.

## 1. Introduction

Parkinson's disease (PD) is a common neurodegenerative disease, second only to Alzheimer's disease (AD) [1]. It is characterized by bradykinesia, rigidity, and tremor, affecting 2%-3% of the population aged more than 65 years [2]. Various epidemiological and experimental studies have shown that old age, environmental risk factors, and genetic factors are related to the pathogenesis of PD. Old age is the leading risk factor for PD [3]. Although PD is rare before 50 years of age, its incidence increases five- to tenfold from the sixth to the ninth decade of life [4].

Recent studies focus on the pathogenesis of PD at both the microscopic biological and macroscopic anatomical levels to find an effective therapy [5]. PD has a variety of pathophysiological mechanisms, including alpha-synuclein aggregate, mitochondrial dysfunction, oxidative stress, calcium homeostasis, axonal transport, and neuroinflammation [6]. The combined action of these mechanisms causes the death of dopaminergic neurons in the substantia nigra, promoting the development of PD.

Despite the noteworthy advances in understanding the etiology and the high-throughput drug screening methods for small molecules, remarkable developments in disease modeling, and improvements in analytical technologies, no therapies are available to prevent the disease from getting worse [7]. More importantly, the current diagnosis of PD depends mainly on the clinical symptoms, and the diagnosis can be confirmed only by autopsy [8]. Therefore, further understanding the molecular mechanism of PD and proposing effective therapeutic targets and treatment programs remain high priorities.

Long noncoding RNA (lncRNA) is a new potential biomarker with biological functions [9]. It has a broad clinical application prospect. Recent studies on lncRNAs have attempted to reveal the mystery of PD. lncRNAs are novel RNA molecules of more than 200 nucleotides in length. They are located in the nucleus or cytoplasm. They play potential roles in regulating the expression levels of protein-coding genes through epigenetic regulation, transcriptional regulation, and posttranslational control, although they hardly or do not encode proteins themselves [10, 11].

lncRNAs function by interacting with three kinds of biomolecules: DNA, RNA, and protein [12]. They form binary and even ternary complexes. Hence, looking for targets interfering with their interactions can help in drug discovery. In the nucleus, they can specifically induce gene silencing on the same or another chromosome by recruiting chromosomal remodeling complexes to specific chromosomal locations [10]. In the cytoplasm, they can serve as competing endogenous RNAs (ceRNAs) to modulate miRNA expression [13–15]. They can also control transcriptional activity by directly or indirectly targeting mRNAs [16].

lncRNAs are highly enriched and expressed in the central nervous system (CNS) [17]. They play a role in neural development and brain evolution through histone modification, transcription cofactors, mRNA decay, and alternative splicing [18], thus mediating behavior and cognition [18, 19].

**Figure 1:**
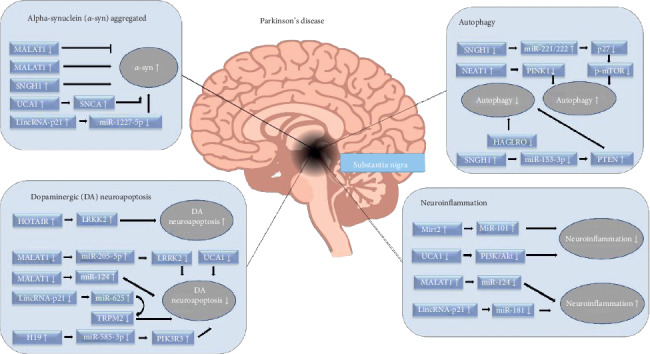
Multiple functions of long noncoding RNAs (lncRNAs) in pathological changes of Parkinson's disease.

The abnormal expression of lncRNAs is closely associated with several human neurological diseases, including PD, AD, Huntington's disease, and schizophrenia [20–23]. Importantly, lncRNA expression changes during aging, thus serving as the major risk factor for the development of PD [24, 25]. Although the research on the role of lncRNAs in PD is very limited at present, the potential regulatory mechanism should not be ignored. In conclusion, lncRNA, as an important endogenous regulatory mechanism in a human body, is expected to become a new therapeutic target to prevent PD from getting worse. This study provided brief, but focused insights into the role of lncRNAs in regulating multilevel activities in PD (Figure 1).

## 2. Aberrant Expression of lncRNAs in PD

Existing studies have confirmed that lncRNAs are highly expressed in various parts of both the CNS and the brain [26, 27]. Several studies have shown the involvement of aberrant lncRNAs in the pathological process of PD (Table 1 and 2). lncRNAs were detected in brain tissues (the cingulate gyrus) of patients with PD. Five significantly differentially expressed lncRNAs were found, including significantly upregulated expression of H19 upstream conserved 1 and 2 and significantly downregulated expression of long intergenic noncoding RNA-p21 (lincRNA-p21), metastasis-associated lung adenocarcinoma transcript 1 (MALAT1), small nucleolar RNA host gene 1 (SNHG1), and trophoblast-derived noncoding RNA (TncRNA) [48]. Patients with early PD show Lewy-related pathology only in brain stem regions. Subsequently, the cingulate gyrus is affected. These significantly differentially expressed lncRNAs are expressed in the cingulate gyrus of patients, indicating that the disorder of lncRNAs may occur in the early stage of PD and has the potential to be used as a biomarker in the early stage of the disease. A recent study found that PD-related genes associated with lncRNAs decreased in the substantia nigra and cerebellum of patients, which was consistent with the results obtained in peripheral blood monocytes [47]. Most of them were detected in the cerebrospinal fluid- (CSF-) derived exosomes, providing the basis for lncRNAs as a potential biomarker of PD. lncRNAs in the CSF of patients with PD have a higher frequency compared with controls, corroborating previous reports that various lncRNAs performed essential functions in the regulation of progression of PD [50]. CSF is close to the main site of PD pathology and is an optimal source of biomarkers for neurodegenerative disorders because of the lack of a barrier between CSF and the brain [57]. lncRNAs in CSF have the potential as a biomarker for PD. In addition, the change in lncRNAs may be one of the patient's symptom severity indexes. In a comparative study, deep brain stimulation could effectively improve the symptoms of patients with PD and change the blood leukocyte cells of 663 lncRNAs [20]. Similarly, PD-related lncRNAs were also found in different PD animal and cell models. The SH-SY5Y cell line stimulated with alpha-synuclein produced 53 upregulated lncRNAs and 69 downregulated lncRNAs compared with the control group [5]. The specific downregulation of AS Uchl1 in iMN9D cell lines stimulated with 1-methyl-4-phenylpyridinium (MPP+) was also verified [28]. Further, 279/164 upregulated lncRNAs and 477/177 downregulated lncRNAs were found in Th-SNCA^*∗*^A30P^*∗*^A53T transgenic mice and alpha-synuclein transgenic mice [53, 55], respectively. In Nrf2 knockout mice, 74 upregulated lncRNAs and 160 downregulated lncRNAs were found [54]. Differential lncRNA expression in conventional nontransgenic PD models has also been studied. A total of 512 lncRNAs related to PD have been identified in a 6-hydroxydopamine (6-OHDA) rat PD model, among which 54 are known lncRNAs [56].

lncRNAs detected in the aforementioned studies were different because of different sampling locations and detection techniques, besides differences between individuals. They play an important role in the pathological process of PD and may serve as a potential diagnostic marker and therapeutic target for PD. Furthermore, they may also be used to quantify the efficacy of medication and surgery in patients. However, obtaining a large number of multicenter databases to quantify lncRNA changes in PD is difficult due to the high cost of sequencing technology. However, with the progress of science and technology, lncRNAs have a great potential as a diagnostic marker and therapeutic target for PD.

## 3. Association of lncRNAs with PD-Linked Genes

Currently, six widely recognized sites, including SNCA (alpha-synuclein), Parkin (PARK2), PINK1 (PARK6), DJ-1 (PARK7), LRRK2 (PARK8), and ATP13A2 (PARK9), can cause hereditary single-gene PD [27]. As early as in 2016, the expression of lncRNA AC079630 and uc001lva.4 (close to the LRRK2 gene locus) was detected in the CSF of patients with PD [50]. In addition, lncRNA HOX Transcript Antisense RNA (HOTAIR) also regulated the expression of LRRK2 [30]. The microtubule-associated protein tau (MAPT) gene, which encodes tau protein, has also been identified as a susceptibility gene in PD [58]. High expression levels of MAPT may lead to an increase in the prevalence of neurodegenerative diseases [59]; the methylation of MAPT promoter is particularly related to PD [60]. In the pathology of PD, MAPT-AS1 can control the progression of the disease by inhibiting the methylation of the MAPT promoter [35]. A recent study reported a number of lncRNAs encompassing transcriptional units in proximity to PD-linked protein-coding genes, including SNCA, LRRK2, PINK1, DJ-1, UCH-L1, MAPT, and GBA1. Also, a correlation was found between the expression profile of each lncRNA and its adjacent coding genes. This study indicated that these lncRNAs might be involved in the pathogenesis of the disease by regulating their adjacent PD-related bases, thus having the potential to be used as biological diagnostic markers and therapeutic targets for PD [47].

## 4. Roles of lncRNAs in PD Pathophysiology

### 4.1. Aggregation of lncRNAs and Alpha-Synuclein in PD

Alpha-synuclein is a protein closely related to neurodegenerative diseases. It is an important part of the Lewy body, and its abnormal aggregation is associated with PD, Lewy body dementia, and multisystem atrophy [61]. It causes abnormal deposition of proteins in the cells of patients [62]. Hence, targeting alpha-synuclein can be a potential therapeutic target for PD [63]. Further, understanding the role of alpha-synuclein is important for understanding PD. The aberrant soluble oligomeric conformations of alpha-synuclein contribute to neuronal death and cellular homeostasis disruption. Multiple lncRNAs are clearly involved in this process. Microarray expression profiling was performed in control nontransgenic and human alpha-synuclein transgenic mice by stimulating alpha-synuclein. The expression of 341 lncRNAs in the transgenic mice was significantly different from that in the control mice [55]. This study proved that lncRNAs were involved in the pathological process of alpha-synuclein-induced PD. The same was the case in *in vitro* cell models. After treating SH-SY5Y cell lines with alpha-synuclein oligomers, the changes in lncRNAs were analyzed using a microarray. The results showed noteworthy changes in a series of lncRNAs, including G046036, G030771, AC009365.4, RPS14P3, CTB-11I22.1, and G007549 [5]. This study verified the involvement of lncRNAs in the pathological process of alpha-synuclein-induced PD at the cellular level *in vitro*. lncRNA MALAT1, also known as NEAT2, was highly expressed in neurons and regulated a number of genes involved in dendritic and synaptic development [64, 65]. The overexpression of MALAT1 upregulated the expression of alpha-synuclein in the brains of mice with PD and vice versa [32]. In addition, the specific overexpression of SNHG1 in the brain of mice also led to the aggregation of alpha-synuclein [42]. The highly expressed lincRNA-p21 aggravated the influence of alpha-synuclein on cells through sponging miR-1277-5p [40].

Three therapeutic views exist on the role of alpha-synuclein in PD: reducing the expression of SNCA by directly silencing or inhibiting its promoter expression, activating autophagy or proteasome to increase protein clearance, and reducing posttranslation-based modification [66]. lncRNAs are closely related to alpha-synuclein, and targeting these lncRNAs to act on alpha-synuclein may serve as a fourth therapeutic viewpoint.

### 4.2. lncRNAs and Autophagy in PD

A dynamic balance exists in the expression of alpha-synuclein in normal individuals. The balance is maintained by the actions of the ubiquitin-proteasome system and the lysosomal autophagy system (LAS). The LAS is more important than the ubiquitinproteasome system in mediating alpha-synuclein degradation in neurons [67]. Age is the biggest risk factor for PD [68]; some toxins such as MPTP may induce the disease symptoms. The reason may be the deterioration of the functions of these two systems with aging [69]. Coincidentally, lncRNA expression also changes with aging. Hence, lncRNAs participate in disease pathology through autophagy in PD. In LAS, chaperone-mediated autophagy and macroautophagy participate in the degradation of alpha-synuclein [67, 70]; lncRNAs also play an important role. The SNCA gene, a susceptibility gene in sporadic PD [71], has been repeatedly reported in recent studies on PD [72–74]. It has the specific function of encoding alpha-synuclein. Its point mutation disrupts the cell homeostasis of dopaminergic neurons and leads to disorders of autophagy, resulting in the abnormal deposition of alpha-synuclein protein in the cytoplasm and further promoting the progression of the disease [75]. lncRNA-UCA1 can upregulate the expression of SNCA to promote the progression of PD [76]. In addition, lncRNA NEAT1 (nuclear paraspeckle assembly transcript 1) has been shown to promote autophagy of dopaminergic neurons by stabilizing PINK1 protein in both *in vivo* and *in vitro* models of PD, thereby alleviating the damage to dopaminergic neurons [38, 76]. The downregulation of SNHG1 promotes the autophagy of dopamine neurons through mir-221/222/p27/mTOR, thus slowing down apoptosis [43]. The overexpression of SNHG1 attenuates autophagy by regulating PTEN/AKT/mTOR signaling pathway in SH-SY5Y cells via sponging miR-153-3p [44].

In conclusion, lncRNAs can be targeted to restore and strengthen the cell homeostasis of patients, maintain the balance of the autophagy system, and further eliminate alpha-synuclein as a potential treatment for PD.

### 4.3. lncRNAs and Apoptosis of Dopaminergic Neurons in PD

The apoptosis of dopaminergic neurons is the characteristic pathological manifestation of PD. Various lncRNAs play different roles in this process. lncRNA HOTAIR was highly expressed in the MPTP-induced PD mouse model and MPP(+)-induced PD cell model. HOTAIR specifically improved the stability of LRRK2 mRNA (LRRK2 mutations are widely recognized as the most common cause of dominant PD, and LRRK2 is one of the risk factors for PD [77]) and upregulated its expression to promote the apoptosis of dopaminergic neurons. Knocking down HOTAIR would inhibit the apoptosis of dopaminergic neurons by reducing the activity of caspase-3 [30]. lncRNA MALAT1 is highly expressed in PD as a ceRNA to regulate miRNA expression. The knockout of MALAT1 in mice inhibited the MPTP-induced apoptosis of dopaminergic neurons by upregulating miR-124 [31, 34]. MALAT1/miR-205-5p axis regulates MPP(+)-induced apoptosis by targeting LRRK2 [33]. Similarly, the knockout of SNHG1 inhibited the MPTP-induced apoptosis of dopaminergic neurons in mice with PD by reducing alpha-synuclein-induced cytotoxicity [42], and H19 attenuates apoptosis through regulating miR-585-3p/PIK3R3 [45]. The downregulation of UCA1 ameliorates the apoptosis of dopaminergic neurons. [46]. Suppression of HAGLRO can also decrease apoptosis and autophagy in both *in vivo* and *in vitro* PD models [29].

Slowing down the progression of the disease by targeting the regulation of these apoptosis-related lncRNAs is a promising therapeutic option.

### 4.4. lncRNAs and Neuroinflammation in PD

Neuroinflammation is regarded as one of the most common contributors to PD [78]. Increasing evidence suggests that inflammation may serve as a crucial player in the death of dopaminergic neurons [79]. Existing studies demonstrated that lncRNAs were responsible for the differentiation of immune cells and corresponding immune response in mammals. When the innate immune system and inflammatory signals are overactivated, a large number of free radicals and proinflammatory cytokines are produced, leading to inflammatory cascade and neurodegeneration, which may be one of the molecular mechanisms of lncRNA involvement in PD pathology [80]. microRNA-124 reduced neuroinflammation in PD, while MALAT1 promoted a neuroinflammatory response in PD through sponging miR-124, leading to the secretion of a large number of proinflammatory factors and promoting the progression of PD [31]. lncRNA myocardial infarction associated transcript 2 (Mirt2) is considered to be a negative feedback medium for the excessive inflammatory response, indicating that Mirt2 can inhibit excessive inflammation [81]. In PD, Mirt2 can sponge miR-101 to reduce the inflammatory response in neuropathology [36]. lincRNA-p21 (3100nt) is located on chromosome six. It is involved in cell proliferation, metabolism, and reprogramming and regarded as a potential diagnostic marker in various diseases [82]. In PD, lincRNA-p21 aggravates the inflammatory response. As stated earlier, the highly expressed lincRNA-p21 inhibits the activity of dopaminergic neurons through sponging miR-1277-5p, thus aggravating the influence of alpha-synuclein on cells [40]. Further studies have shown that lincRNA-p21, as a ceRNA, constitutes an lncRNA-miRNA-mRNA regulatory network that indirectly acts on mRNA to exert its effect. For example, specifically knocking down lincRNA-p21 in PD reduces MPP(+)-induced neuronal damage by regulating the mir-625/TRPM2 axis [39]. Downregulation of lncRNA UCA1 could reduce oxidative stress and inflammation through the inhibition of the PI3K/Akt signaling pathway [46]. The central inflammation mechanism mediated by the excessive activation of microglia is also one of the important initiating factors for the development of PD [83]. The overexpressed lincRNA-p21 in PD regulates the activation of microglia through the feedback loop formed with the miR-181 family to promote the occurrence of neuroinflammation and the development of the disease [41].

## 5. lncRNAs as Putative Biomarkers and Therapeutic Targets for PD

Increasing scientific data show that certain lncRNAs alter differentially over time in the brains of patients with PD [48]. As the symptoms of Parkinson's disease improve, the level of lncRNA and microRNA also changes [20, 84]. At the same time, a recent study showed that lnc-MKRN2-42: 1 in PD patients was positively correlated with MDS-UPDRS III score [52]. CSF is very close to the main site of PD pathology. As molecular changes in the PD patient's brain are reflected in CSF composition, the CSF represents an optimal source of biomarkers of PD. lncRNAs in CSF have the potential as a biomarker for PD because of the lack of a barrier between CSF and the brain. The changes in lncRNA expression levels in blood leukocyte samples may also be related to disease status [20]. It has been demonstrated by researchers that blood leukocytes can serve as a feasible and reliable tissue source to test for disease-induced and treatment-related transcript changes [85]. lncRNAs have a higher abundance than protein-coding genes, so more regulation of lncRNA expression can be observed in the same sample, which provides a greater possibility for examining lncRNA-based biomarkers. Large sizes of lncRNAs can fold into complex secondary/tertiary structures and scaffolds, through which they may interact with various proteins, transcriptional regulators, mRNA, and DNA sequences [86]. This may be associated with the initiation and progression of PD. The existence of a large amount of regulation of lncRNA interaction sites for development based on the structure of the new PD drugs provides a broader platform. In addition, considering that lncRNA is involved in multiple cell signal transduction pathways in PD, it can be used to formulate specific PD diagnosis and targeted therapy strategies. The potential mechanisms of action of lncRNAs include the inhibition of the expression of PD-linked genes, reduction in the production of alpha-synuclein, maintenance of autophagy system balance, delay in the apoptosis of dopaminergic neurons, alleviation of nerve inflammation, and so forth. All these findings indicate that lncRNAs have the potential to become a putative biomarker for PD. lncRNAs are expected to play an important role as a biomarker and therapeutic target for the early detection of PD.

Although the research of lncRNA as a biomarker in PD is still in its infancy, it is exciting that, in certain research areas, clinical trials have started on lncRNA as a biomarker [87–90]. In addition, circulating HOTAIR can be used in the diagnosis of breast cancer [91]. HOTAIR can be used to predict the recurrence of HCC [92]. MALAT1, UCA1, ANRIL, and NEAT1 can be used to predict early and metastatic lung cancer [93]. UCA1, H19, and HOTAIR can be used as biomarkers to detect bladder cancer [94]. All these indicate that lncRNA also has the potential to become a new diagnostic and prognostic marker for PD.

As the expression level of lncRNA in PD is related to the initiation and progression of PD and its symptoms, it can be used as a potential therapeutic target for PD. It is possible to target lncRNA to regulate its expression in a variety of ways. For example, the use of lncRNA-specific siRNA, such as the downregulation of siRNA-mediated MALAT1 expression can inhibit MPP(+)-induced apoptosis of DA neurons [33]. In general, the therapy based on lncRNA as a biomarker and possible therapeutic target for PD is still promising.

## 6. Conclusions

The research on lncRNAs is in the initial stages. An increasing number of studies have been conducted on the role of lncRNAs in PD in the last three years. Some studies have shown that lncRNAs are involved in the initiation and progression of PD. A large number of lncRNAs have been found to provide a new basis for the development of early diagnosis and treatment of PD, and the expression of lncRNAs can also be used to predict the symptoms of PD patients. Previous studies have found that some lncRNAs play a protective role in PD (such as UCHL1, MAPT-AS1, and Mirt2), and some of them aggravate the disease progression (such as HOTAIR, MALAT1, NEAT1, lincrna-p21, and SNHG1). Now, many challenges in the study of lncRNAs cannot be ignored. For example, lncRNAs do not have a uniform nomenclature. Compared with coding genes, lncRNAs account for a small proportion, and it is difficult to determine the role of lncRNAs according to nucleotide sequences [95]. Research on lncRNA in PD is predicted to gain popularity in the future. Although researchers have identified some lncRNAs involved in PD using next-generation sequencing, the molecular mechanism of action of these lncRNAs still needs further verification. The sequencing technology is expensive. Moreover, the existing database is not sufficient to quantify the role of lncRNAs in PD. To verify the potential of lncRNAs in PD diagnosis and therapy, it is important to characterize each lncRNA in detail, such as the structure and function of each lncRNA, and to quantify the role of lncRNA in PD in multicenter studies. In the foreseeable future, early screening for PD may be more accurate, thanks to the studies on the lncRNA mechanism, thus improving the efficacy and accuracy of treatment.

## Figures and Tables

**Table 1:** LncRNAs: their mechanism of action in Parkinson' disease.

lncRNA	Tissue/model	Regulation	Pathway targeted by the lncRNAs	References
AS UCHL1	(1) MN9D cells treated with MPP+	Down	AS Uchl1 RNA, as a component of Nurr1-dependent gene network and target of cellular stress, extended the understanding on the role of antisense transcription in the brain	[28]
	(2) DA neurons from PD model treated with MPP+			

HAGLROS	MPTP-induced PD mice and SH-SY5Y cells treated with MPP+	Up	Suppression of HAGLRO decreased apoptosis and autophagy in both *in vivo* and *in vitro* PD models	[29]
			HAGLRO negatively regulated miR-100 expression	
			Suppression of HAGLROS alleviated MPP(+)-intoxicated SH-SY5Y cell injury by activating PI3K/AKT/mTOR pathway	

HOTAIR	SH-SY5Y cells treated with MPP+	Up	With HOTAIR overexpression in SH-SY5Y cells, the expression of LRRK2 increased compared with that in the control	[30]
			HOTAIR knockdown provided protection against MPP(+)-induced DA neuronal apoptosis by repressing caspase 3 activity	

MALAT1	MPTP-induced PD mice and SH-SY5Y cells treated with MPP+	Up	MALAT1 knockdown attenuated MPTP-induced apoptosis of DA neurons in MPTP-induced PD mouse model	[31]
			MALAT1 interacted with miR-124 to negatively regulate its expression	
	MPTP-induced PD mice and SH-SY5Y cells treated with MPP+	Up	MALAT1 was associated with a-synuclein, leading to the increased stability of a-synuclein and its expression	[32]
	MPTP-induced PD mice and MN9D cells treated with MPP+	Up	MALAT1/miR-205-5p axis regulates MPP(+)-induced apoptosis in MN9D cells by targeting LRRK2	[33]
	MPTP-induced PD mice and SH-SY5Y cells treated with MPP+	Up	MALAT1 knockdown attenuated MPP(+)-induced apoptosis of DA neurons in SH-SY5Y cells	[34]
			MALAT1 regulates DAPK1 expression by targeting miR-124-3p	

MAPT-AS1	Brain tissue samples (10 patients with PD and 10 controls)	Down	MAPT-AS1 and DNMT1 have been identified as potential epigenetic regulators of MAPT expression in PD	[35]

Mirt2	SY5Y cells treated with TNF-*α*	Down	Mirt2 exhibited anti-inflammatory properties through miR-101 suppression	[36]
			Mirt2 blocked TNF*α*-triggered NF-*κ*B/p38MAPK pathway	

NEAT1	MPTP-induced PD mice and SH-SY5Y cells treated with MPP+	Up	NEAT1 knockdown promoted cell viability and suppressed cell apoptosis	[37]
			Downregulation of NEAT1 also decreased the ratio of Bax/Bcl-2, the activity of caspase-3, as well as the expression of *α*-synuclein	
	MPTP-induced PD mice and SH-SY5Y cells treated with MPP+	Up	NEAT1 positively regulated the protein level of PINK1 through inhibition of PINK1 protein degradation	[38]
			NEAT1 knockdown could effectively suppress MPTP-induced autophagy that alleviated dopaminergic neuronal injury	

lincRNA-p21	SH-SY5Y cells treated with MPP+	Up	lincRNA-p21 regulated MPP(+)-induced neuronal injury by sponging miR-625 and upregulating TRPM2 in SH-SY5Y cells	[39]
	MPTP-induced PD mice and SH-SY5Y cells treated with MPP+	Up	lincRNA-p21 sponged miR-1277-5p and indirectly increased the expression of *α*-synuclein to suppress viability and activate apoptosis in SH-SY5Y cells	[40]
	MPTP-induced PD mice and SH-SY5Y cells treated with a CM transfer system were used to determine the impact of LPS-treated BV2 cells	Up	p53/lincRNA-p21, together with miR-181/PKC-*δ*, formed a double-negative feedback loop that facilitated sustained microglial activation and the deterioration of neurodegeneration	[41]

SNHG1	MPTP-induced PD mice and SH-SY5Y cells treated with MPP+	Up	SNHG1 could directly bind to miR-15-5p and repress miR-15-5p expression	[42]
			Upregulation of miR-15b-5p alleviated *α*-synuclein aggregation and apoptosis by targeting SIAH1	
			SNHG1 knockdown inhibited *α*-synuclein aggregation and *α*-synuclein-induced apoptosis	
	MN9D cells treated with MPP+	Up	SNHG1 could competitively bind to the miR-221/222 cluster and indirectly regulate the expression of p27/mTOR	[43]
	SH-SY5Y cells treated with MPP+	Up	SNHG1 overexpression lowered viability and enhanced apoptosis in MPP(+)-treated SH-SY5Y cells.	[44]

H19	MPTP-induced PD mice and human neuroblastoma cells treated with MPP+	Down	H19 attenuates apoptosis in MPTP-induced Parkinson's disease	[45]
			H19/miR-585-3p axis regulates MPP(+)-induced apoptosis in human neuroblastoma cells cells by targeting PIK3R3	

UCA1	6-OHDA-induced PD rat	Up	Downregulation of lncRNA UCA1 ameliorates the damage of dopaminergic neurons, reduces oxidative stress and inflammation in PD rats	[46]
			Downregulation of lncRNA UCA1 inhibits the PI3K/Akt signaling pathway.	

**Table 2:** Altered lncRNAs in Parkinson' Disease.

Tissue	Ethnicity/population	LncRNA	Regulation	References
Substantia nigra and cerebellum (9 patients with PD and 8 controls)	Not mentioned (the tissue were obtained from The Netherlands brain Bank)	AK127687, AX747125, GBAP1, SNCA-AS1, UCHL1-AS1, PINK1-AS1, and MAPT-AS1	Up	[47]
Anterior cingulate gyrus (20 patients with PD and 10 controls)	Not mentioned (the tissue were provided by the Neurobiobank Munich (NBM))	H19 upstream conserved 1 and 2	Up	[48]
		LincRNA-p21, MALAT1, SNHG1, and TncRNA	Down	
Exosomes isolated from CSF (47 patients with PD and 27 controls)	Not mentioned (the tissue were provided by the sir Run Run shaw hospital, affiliated with school of medicine, Zhejiang University)	RP11-462G22.1 and PCA3	Up	[49]
Extracellular RNAs present in CSF (27 patients with PD and 30 controls)	Not mentioned (the tissue were provided by the hospital Universitario Donostia, San Sebastian, Spain (MDUD))	AC079630 and UC001lva.4 (close to the LRRK2 gene locus)	Up	[50]
Substantia nigra (11 patients with PD and 14 controls)	Not mentioned (the volunteers who provided the tissue were from USA, UK, Israel, and Germany)	AL049437, U79277, AF052141, AK021454, BC018626, AF147723, AK001884, AY365119, BC151247, BC151234, AK311445, AK310272, AK094351, AF007131, AF119861, CR619166, AK023852, AK074162, AF052176, BC007937, AK025388, AK022431, CR618512, AK021912, AL109681, AF090884, AL359578, AF070543, AK021798, AK024568, U94902, AK024381, AF090910, BC002644, BC064478, AF007141, M28219, AK001998, BC002821, AL049328, AK024684, and AK000420	Up	[51]
		AK024198, AK025097, AK024214, AF052148, AF070579, AK023918, AK022167, AK024938, AL109707, BC000988, AK025271, AL109705, AJ001873, BC029383, AK025360,	Down	
Blood leukocytes sample (3 patients with PD)	Not mentioned	RP11-101C11.1, U1^*∗*^‘#, RP11-425i13.3, RP11-124n14.3, RP11-79p5.3, RP11-462g22.1, RP13-507P19.2, and PCA3	Up	[20]
		RP11-409K20.6, RP4-705O1.1, AC004744.3, RP11-533O20.2, and RP11-542K23.9	Down	
Brain tissue samples (10 patients with PD and 10 controls)	Caucasian descent (the tissue were provided by the Sydney Brain Bank and the NSW Tissue Resource Centre)	MAPT-AS1	Down	[35]
Plasma samples (32 patients with PD and 13 controls)	Not mentioned (the volunteers who provided the tissue were from Beijing Tiantan Hospital)	MSTRG.242001.1, MSTRG.169261.1	Up	[52]
		MSTRG.336210.1, lnc-MKRN2-42 : 1	Down	
Substantia nigra from mice (3 Nrf2+/+ mice and 3 Nrf2-/- mice)		AK020441, AK020330, NR_003555, NR_073442, AK040987, ENSMUST00000142871, ENSMUST00000153819, ENSMUST00000132304, uc011ysu.1, and so forth (a total of 74)	Up	[53]
		ENSMUST00000139383, NR_024325, AK047372, ENSMUST00000156693, ENSMUST00000181307, AK076880, AK036620, TCONS_00017218, TCONS_00022981, TCONS_00004085, and so forth (a total of 160)	Down	
Whole mesencephalic tissues from mice (6 *α*-synuclein transgenic C57BL/6 mice and 6 control mice)		uc.44-, BC037523, and so forth (a total of 164)	Up	[54]
		uc.12+, AK076860, and so forth (a total of 177)	Down	
The striatum from rat (9 PD model and 9 control rats)		XLOC_026924, XLOC_029397, XLOC_004631, XLOC_005439, XLOC_018657, XLOC_016191, XLOC_022926, AABR07029901.1, XLOC_025867, XLOC_016202, and so forth (a total of 451)	Up	[55]
		XLOC_028318, XLOC_037769, XLOC_029657, XLOC_010572, XLOC_017775, XLOC_018598, Rn50_5_1638.1, XLOC_006399, AABR07027137.1, XLOC_001547, and so forth (a total of 61)	Down	
SY-SH5Y cells treated with a-synuclein oligomers		A total of 53 lncRNAs	Up	[56]
		A total of 69 lncRNAs	Down	
Whole mesencephalic tissues from mice (6 *α*-synuclein transgenic C57BL/6 mice and 6 control mice)		uc.44-, BC037523, and so forth (a total of 164)	Up	[5]
		uc.12+, AK076860, and so forth (a total of 177)	Down	
